# Supplementary material for: Immunosuppressive regimens and long-term kidney transplant outcomes: A dual survival modeling framework
Source: PLoS One. 2026 Jun 26;21(6):e0339109. doi: 10.1371/journal.pone.0339109 (PMC13309038; doi:10.1371/journal.pone.0339109)
Supplement: S1 Checklist — (DOCX) [file pone.0339109.s004.docx]

**STROBE Statement Checklist for Cohort Studies**

Manuscript Title:
Immunosuppressive regimens and long-term kidney transplant outcomes: a dual survival modelling framework

| Item | Recommendation | Reported in Manuscript |
| --- | --- | --- |
| 1a | Indicate study design in title or abstract | Abstract; Methods |
| 1b | Informative summary in abstract | Abstract |
| 2 | Scientific background and rationale | Introduction |
| 3 | Objectives and hypotheses | Final paragraph of Introduction |
| 4 | Key elements of study design | Methods – Study Design |
| 5 | Setting, locations, dates, follow-up | Methods – Data Source and Cohort Selection |
| 6a | Eligibility criteria and participant selection | Methods – Study Population |
| 7 | Definitions of outcomes, exposures, confounders | Methods – Variables and Outcomes |
| 8 | Data sources and measurement | Methods – Data Source and Variable Definitions |
| 9 | Efforts to address bias | Statistical Analysis; Discussion – Limitations |
| 10 | Study size determination | Methods – Cohort Description |
| 11 | Handling of quantitative variables | Statistical Analysis |
| 12a | Statistical methods and confounding control | Statistical Analysis |
| 12b | Subgroup/interactions | Survival Analyses and ML Modeling |
| 12c | Missing data handling | Methods – Missing Data Handling |
| 12d | Loss to follow-up | Not applicable; registry follow-up and censoring mechanisms described in Methods – Study Outcome |
| 12e | Sensitivity analyses | Statistical Analysis / Supplementary Analyses |
| 13a | Participant numbers at each stage | Results; Figure 1 |
| 13b | Reasons for non-participation | Registry-based cohort; exclusions described in Methods |
| 13c | Flow diagram | Figure 1 |
| 14a | Participant characteristics | Table 1 |
| 14b | Missing data summary | Methods – Missing Data Handling |
| 14c | Follow-up time summary | Results – Survival Outcomes |
| 15 | Outcome events over time | Results; Kaplan–Meier Analyses |
| 16a | Main estimates and precision | Tables 2–4 |
| 16b | Categorization boundaries | Methods – Variable Definitions |
| 16c | Absolute risk estimates | Not applicable; survival probabilities at 1, 3, 5, and 10 years are reported instead (Results – Survival Outcomes) |
| 17 | Other analyses/subgroups/sensitivity analyses | Results – ML Survival Models and Sensitivity Analyses |
| 18 | Summary of key results | Discussion – Paragraph 1 |
| 19 | Limitations and bias | Discussion – Limitations |
| 20 | Overall interpretation | Discussion |
| 21 | Generalisability | Discussion |
| 22 | Funding source and role | Funding Statement |
