# Supplementary material for: Immunosuppressive regimens and long-term kidney transplant outcomes: A dual survival modeling framework
Source: PLoS One. 2026 Jun 26;21(6):e0339109. doi: 10.1371/journal.pone.0339109 (PMC13309038; doi:10.1371/journal.pone.0339109)
Supplement: Attachment — Submitted filename: Response_to_Reviewers.docx [file pone.0339109.s006.docx]

**Response to Reviewers**

**Manuscript ID:** PONE-D-25-64299
**Title (Revised):** Immunosuppressive Regimens and Long-Term Kidney Transplant Outcomes: A Dual Survival Modeling Framework
**Journal:** PLOS ONE

Dear Dr. Bhimma and Reviewers,

We sincerely thank the Academic Editor and Reviewers for their thoughtful and constructive feedback. We have carefully revised the manuscript to address all comments and journal requirements. All changes have been incorporated into the revised manuscript and highlighted in the tracked-changes version.

Below, we provide a detailed point-by-point response.

**Journal Requirements**

**Requirement 1:**

***Please ensure that your manuscript meets PLOS ONE's style requirements, including those for file naming.***

**Response:**
The manuscript has been revised to fully conform with PLOS ONE formatting and file-naming requirements using the journal’s provided templates. Section headings, abbreviations, tables, and figure captions were updated to align with PLOS ONE style guidelines.

**Requirement 2:**

***We note that you have indicated that there are restrictions to data sharing for this study. PLOS only allows data to be available upon request if there are legal or ethical restrictions on sharing data publicly.***

***Before we proceed with your manuscript, please address the following prompts:***

***a) If there are ethical or legal restrictions on sharing a de-identified data set, please explain them in detail (e.g., data contain potentially identifying or sensitive patient information, data are owned by a third-party organization, etc.) and who has imposed them (e.g., a Research Ethics Committee or Institutional Review Board, etc.). Please also provide contact information for a data access committee, ethics committee, or other institutional body to which data requests may be sent.***

***b) If there are no restrictions, please upload the minimal anonymized data set necessary to replicate your study findings to a stable, public repository and provide us with the relevant URLs, DOIs, or accession numbers.***

***You also have the option of uploading the data as Supporting Information files, but we would recommend depositing data directly to a data repository if possible.***

***We will update your Data Availability statement on your behalf to reflect the information you provide.***

**Response:**
We thank the editors for this request. The dataset analyzed in this study was obtained from the Organ Procurement and Transplantation Network (OPTN), operated by the United Network for Organ Sharing (UNOS). These data are owned by a third-party organization and contain sensitive, de-identified patient-level information subject to strict data use agreements that prohibit public redistribution.

Accordingly, the minimal dataset cannot be shared publicly. However, qualified researchers may request access directly from UNOS by submitting a formal data request and executing a data use agreement (<https://unos.org/data/>). The Data Availability Statement has been revised to reflect this information.

**Requirement 3:**

***If the reviewer comments include a recommendation to cite specific previously published works, please review and evaluate these publications to determine whether they are relevant and should be cited. There is no requirement to cite these works unless the editor has indicated otherwise.***

**Response:**
All reviewer-suggested references were evaluated for relevance. Terminology updates consistent with contemporary ISN and KDIGO nomenclature guidance (Am J Transplant. 2021;21:901–902) were incorporated where appropriate.

**Additional Editor Comments**

**Editor Comment 1**

The imputation strategy—particularly imputing missing cPRA values as zero with a binary indicator—may introduce bias and should be more explicitly justified.

**Response:**
We thank the editor for this important methodological observation. The Methods section (Predictor Variables and Data Preprocessing) has been revised to clarify the rationale for imputing missing cPRA values as 0 with a binary missingness indicator.

Specifically, we now note that the empirical distribution of cPRA demonstrated a median value of 0%, consistent with registry-wide OPTN reporting patterns. This reflects historical registry structure, in which cPRA was not consistently captured in earlier transplant eras and unsensitized recipients frequently had values recorded as zero or missing due to evolving reporting practices. The imputation approach was therefore selected to preserve cohort size while allowing the multivariable models to account for potential non-random missingness through inclusion of a missingness indicator.

We also acknowledge the potential for attenuation of sensitization-related associations and have clarified this limitation in the manuscript. These revisions improve transparency regarding the rationale, empirical justification, and limitations of the imputation strategy.

**Editor Comment 2**

While the authors appropriately use multivariable adjustment, the conclusions regarding “protective” or “beneficial” effects of specific induction and maintenance regimens are sometimes phrased too strongly for a retrospective registry-based analysis. Confounding by indication (e.g., sicker or higher-immunologic-risk patients preferentially receiving ATG or steroid-sparing regimens) is not adequately addressed. Consideration of sensitivity analyses (e.g., propensity-based methods) or clearer acknowledgement of this limitation would strengthen the work.

**Response:**
We thank the editor for this important comment. Language throughout the Abstract, Results, Discussion, and Conclusion has been revised to avoid causal interpretations. Terms such as “protective” or “beneficial” were replaced with association-based phrasing. The Strengths and Limitations section was expanded to explicitly acknowledge confounding by indication and non-randomized treatment allocation. Findings are now framed as adjusted observational associations rather than causal effects.

**Editor Comment 3**

The study spans more than two decades (2000–2024), during which transplant practices, immunosuppressive protocols, allocation systems, and outcomes have changed substantially. Although the sample size is impressive, the lack of explicit adjustment for transplant era or policy changes (e.g., allocation system revisions) is a concern and may affect the interpretation of long-term outcomes.

**Response:**
We thank the editor for highlighting the importance of accounting for temporal changes in transplant practice. We have revised the statistical modeling strategy to explicitly adjust for transplant era (2000–2004, 2005–2009, 2010–2014, 2015–2019, 2020–2024). The Methods section has been updated to clarify this adjustment and to note that the final era category captures contemporary transplant practice, including recent allocation system updates. Sensitivity analyses without era adjustment are now provided in S1 and S2 Tables, demonstrating stable effect estimates and supporting the robustness of the primary findings. These revisions are described in the Methods and reflected in the revised Results and Supporting Information tables.

**Editor Comment 4**

The findings regarding racial and ethnic differences, donor race effects, and induction regimen combinations (e.g., ATG + IL-2R) are potentially sensitive and clinically important. These results merit more cautious interpretation and contextualization to avoid overinterpretation of registry-based associations

**Response:**
The Discussion has been revised to provide additional contextualization. Race and ethnicity are now described as registry-reported social descriptors rather than biologic determinants, and interpretations emphasize structural and allocation-related factors. Induction regimen findings were reframed to highlight treatment selection rather than pharmacologic causation.

**Editor Comment 5**

Please update terminology in line with the lastest ISN guidelines - see Am J Transplant. 2021;21:901–902.

**Response:**
Terminology has been updated throughout the manuscript consistent with ISN/KDIGO guidance, including replacing “end-stage renal disease” with “kidney failure” where appropriate.

**Reviewer #1**

We thank Reviewer #1 for the positive and encouraging evaluation of our work.

**Reviewer #2 — Specific Comments**

**Comment:**

Although the title talks about the precision immunosuppression there is no mention of use of pharmacometrics, genetic testing (e.g., CYP3A5) or any biomarkers to achieve superior graft/patient outcome. Suggest changing the title and removing precision immunosuppression from it.

**Response:**
We appreciate this constructive suggestion. To better reflect the scope of the registry-based analysis and avoid implying pharmacogenomic or biomarker-guided therapy, the title has been revised to:

**“Immunosuppressive Regimens and Long-Term Kidney Transplant Outcomes: A Dual Survival Modeling Framework.”**

**Comment:**

***Line no.222- 5 year death censored graft survival is mentioned 80% but it is mentioned 75.8% at 4 years median (table 1). Please explain the discrepancy***

**Response:**
The apparent discrepancy reflects the difference between follow-up status metrics and Kaplan–Meier survival estimates. The 75.8% value represents censored observations at median follow-up, whereas the 80.1% value reflects Kaplan–Meier 5-year survival probability. To clarify this distinction:

- The Table 2 footnote now states that events and censoring represent follow-up status rather than survival estimates.
- The Table 3 caption was revised to specify that survival values are derived from time-to-event analysis.

**Comment:**

*What was the difference between KDPI of DCD vs non-DCD donors?*

**Response:**
A descriptive comparison has been added to the Results section indicating that DCD donors had higher KDPI compared with non-DCD donors (median [IQR] 50% [33–68] vs 37% [17–60]; Mann–Whitney p<0.001). Detailed distributions are now presented in S3 Table.

**Comment:**

***Please provide details of incompatible transplants in each group.***

**Response:**
The registry variables included in the analytic dataset did not contain complete or consistently available measures of immunologic incompatibility, such as ABO incompatibility, crossmatch status, or desensitization protocols suitable for stratified analysis. Consequently, subgroup analyses based on incompatibility could not be performed. Calculated panel-reactive antibody (cPRA) was included as a covariate to partially account for sensitization risk. A statement acknowledging this limitation has been added to the Strengths and Limitations section to ensure appropriate interpretation of registry-based findings.

**Comment:**

***Combination therapy with ATG and IL2R had worst graft survival. What could be the reason for this?***

**Response:**
The Discussion has been revised to clarify that the observed association may reflect treatment selection for recipients with greater immunologic complexity or higher baseline risk rather than a direct pharmacologic effect of dual-antibody therapy. This interpretation emphasizes confounding by indication and aligns with the observational study design.

**Closing Statement**

We thank the Academic Editor and Reviewers again for their insightful feedback, which has substantially strengthened the clarity, methodological rigor, and interpretive balance of this manuscript. We hope that the revisions adequately address all concerns and respectfully submit the revised manuscript for your consideration.

Sincerely,
**Kunle Apanisile, PhD**
(on behalf of all authors)
