# Supplementary material for: Immunosuppressive regimens and long-term kidney transplant outcomes: A dual survival modeling framework
Source: PLoS One. 2026 Jun 26;21(6):e0339109. doi: 10.1371/journal.pone.0339109 (PMC13309038; doi:10.1371/journal.pone.0339109)
Supplement: Attachment — Submitted filename: Response to Reviewers.docx [file pone.0339109.s007.docx]

**Response to the Editor and Reviewers**

**Manuscript Title:** Immunosuppressive regimens and long-term kidney transplant outcomes: a dual survival modelling framework
**Manuscript ID:** PONE-D-25-64299R1

Dear Dr. Bhimma,

We sincerely thank you and the reviewers for the careful evaluation of our manuscript and for the constructive feedback provided throughout the review process. We are grateful that the reviewers found the manuscript technically sound and the statistical analyses appropriate. We have carefully revised the manuscript to further improve clarity, reporting transparency, and consistency with PLOS ONE publication standards.

Response to Reviewer #2

We thank Reviewer #2 for the positive assessment of the revised manuscript and for confirming that the prior comments had been satisfactorily addressed. We appreciate the reviewer’s evaluation that the manuscript is technically sound, clearly written, and supported by appropriate statistical analyses. As no additional scientific revisions were requested, the current revision focused primarily on reporting compliance, clarity, and final editorial refinement.

The following updates were made in the revised manuscript:

1. Data Availability Statement revised
   The Data Availability Statement was updated to clarify that the study utilized third-party registry data obtained from the Organ Procurement and Transplantation Network (OPTN), operated by the United Network for Organ Sharing (UNOS). We clarified that the authors are unable to publicly share the data because of data use restrictions and specified the process by which qualified researchers may request access through UNOS. We also added the public GitHub repository containing the analytical code used for data preprocessing, survival modeling, and generation of tables and figures.
2. Abstract refined
   The abstract was revised for improved clarity, concision, consistency of terminology, and appropriate abbreviation usage while preserving all substantive findings and statistical results.
3. STROBE reporting compliance
   A completed STROBE checklist for cohort studies has been uploaded as supplementary material (S4 Checklist). In addition, a statement was added to the Methods section indicating that the study was reported in accordance with the STROBE reporting guideline for observational cohort studies.
4. Minor wording and consistency corrections
   Minor wording inconsistencies identified during the final manuscript review were corrected, including clarification of Kaplan–Meier survival descriptions, refinement of selected Discussion section phrasing, and standardization of statistical notation and terminology throughout the manuscript.
5. Formatting and figure review
   The manuscript, tables, figures, supplementary materials, and references were carefully reviewed and revised to ensure consistency with PLOS ONE formatting and figure preparation guidelines.

We believe that these revisions have strengthened the manuscript and addressed all remaining editorial considerations. We appreciate the time and effort invested by the editor and reviewers throughout the review process and respectfully submit the revised manuscript for your consideration.

Sincerely,

**Kunle Apanisile, PhD**
on behalf of all authors
